# The outbreak of acute gastroenteritis caused by sapovirus at a school in Shenzhen, China, 2023

**DOI:** 10.3389/fpubh.2025.1572482

**Published:** 2025-05-09

**Authors:** Ji Li, Zhenyu Huang, Chucuan Wu, Min Zhang, Hongxiong Guo, Yuan Li

**Affiliations:** ^1^Shenzhen Bao'an District Center for Public Health Services, Shenzhen, China; ^2^Shenzhen Bao'an District Center for Disease Control and Prevention, Shenzhen, China; ^3^Heyuan Yuanchen District Center for Disease Control and Prevention, Heyuan, China; ^4^Jiangsu Provincial Center for Disease Control and Prevention, Nanjing, China

**Keywords:** sapovirus, airborne transmission, prevention strategies, cohort study, outbreak

## Abstract

**Background:**

Between February 6 and 8, 2023, an increasing number of students showed symptoms of vomiting in a school in Shenzhen, China. To identify the cause of this outbreak and curb disease spread on February 9, 2023, and an outbreak investigation including a case-control study was conducted.

**Methods:**

A structured questionnaire was used to collect the symptoms of all cases, a retrospective cohort study were conducted to examine the risk factors for diarrhea or vomiting. To find the contamination source, we investigated the environment all buildings in schools. Relative risk was presented and Chi-square test was performed. All the analyses were performed with OpenEpi software version 2.3.1 online. PCR was used to test stool specimens.

**Results:**

This outbreak was caused by sapovirus, and lasted for 9 days. 70.9% of cases reported vomiting, 53.5% diarrhea, 38.4% bellyache, 14.0% feel nauseous, and 69.6% had diarrhea no more than twice. Eating food provided by the school or drinking water from direct-drinking machine was not associated with the acquisition of AGE based on univariate analysis. The students who had passed near a spot of vomit (<2.4 m) were more likely to get AGE with a relative risk of 5.09 (95%CI: 2.58, 10.04). The case number with AGE in classrooms using standard operating procedure for vomit cleanup is obviously lower than that in ones not handling vomit according to the standard procedure [relative risk (RR):0.34, 95%CI: 0.15, 0.77].

**Conclusion:**

Sapovirus was the causative agent of this AGE outbreak, and airborne transmission was the primary route of infection. Prompt decontamination and the use of vomit bags significantly reduce the incidence of AGE while close contact with contaminated materials increases the risk of infection. These measures should be prioritized in public health strategies to prevent and control viral gastroenteritis outbreaks.

## Introduction

Sapovirus, a member of the caliciviridae family, is an important agent in acute gastroenteritis (AGE) (1). In developing countries and regions with limited resources, it accounts for 3–17% of acute viral gastroenteritis cases (2–5). However, studies from Asian countries such as Japan identified sapovirus as the leading cause of AGE, it is the third most common virus after norovirus and rotavirus (6). Sapovirus-induced gastroenteritis is typically milder than norovirus, and severe cases and fatalities among them are rare. Nonetheless, the AGE outbreaks caused by sapovirus in childcare facilities, nursing homes, and schools remain a significant public health concern (7). Although norovirus outbreaks in public places in China are well-documented (8, 9), there is a paucity of research on sapovirus, another member of Caliciviridae family, as a cause of AGE.

Fecal-oral transmission is the primary route of human-to-human sapovirus transmission. While foodborne transmission, particularly in crowded households, is common, sapovirus can also spread through contact with vomit or contaminated surfaces (5). Direct or indirect contact with contaminated water, food, objects, or environmental surfaces can facilitate transmission (10). Some studies suggest that sapovirus may be even more transmissible than norovirus, as lower infectious dose estimates indicate (11). In recent years, while some studies have suggested airborne transmission as a significant route of transmission in sapovirus outbreaks, most studies of them lack sufficient epidemiological evidence to support this claim (12, 13).

Despite the frequent reporting of acute viral gastroenteritis in China, cases caused by sapovirus are relatively rare. Molecular surveillance shows that sapovirus ranks the fourth agent causing AGE in Guangdong (14), a bustling metropolis in southeastern China's Pearl River Delta with a total of 18,000,000 population. At a school in Bao'an district of Shenzhen city, from February 6 to 8, 2023, an increasing number of students showed symptoms of vomiting. Shenzhen Bao'an Center for Disease Control and Prevention (SBCDC) was invited to identify the cause of this outbreak and curb disease spread on February 9, 2023, and an outbreak investigation including a case-control study was conducted subsequently.

## Materials and methods

### Setting

**Figure 1:**
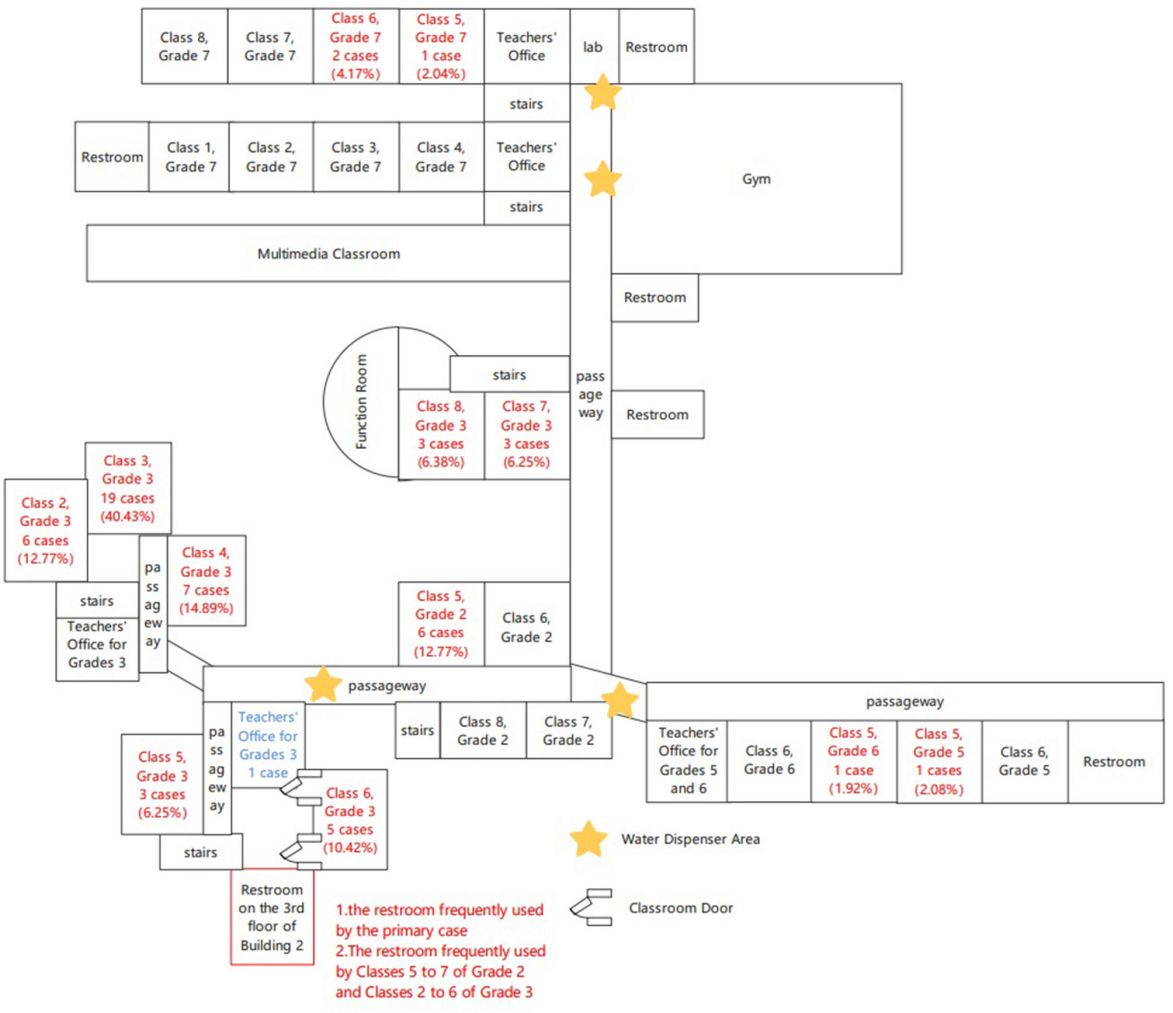
The diagram of the buildings at the school, Bao'an district, Shenzhen, China. Red indicates the classroom with cases.

This school has seven buildings in the campus of 38,760 square meters. Five out of them are teaching buildings accommodating 68 classrooms (shown in Figure 1). It has 3,251 students including 2,111 elementary school students and 1,140 junior high school students, 273 teachers and 62 employees. There is a cafeteria next to the teachers' dormitory, which is exclusively for teachers and staff, while students rely on ready-to-eat meals delivered by external caterers. The tab water used in this school is sourced from the municipal water supply system, which is treated by a direct-drinking machine before being provided to students and staff. The direct-drinking water was tested on February 7 of 2023, and the results demonstrated that the water follows the specified standards.

### Epidemiological investigation

One child (child X) at the third-grade class 3 reported that she had the onset of diarrhea and vomit since at 5:00 a.m. on February 6 about 12 h after eating frozen dumplings with abnormal taste bought from a supermarket. She did not take any medicine due to symptoms gradually subsiding and then went to school regularly on February 7. She went to the washing room located at the third floor of building 2 and had diarrhea twice at 9:30 am and 12:00, respectively. She was picked home at 12:30. Although she had AGE which might be caused by an infectious agent, her vomit and diarrhea were not handled as infectious excrement timely. The location of the washing room she used is indicated as Figure 1. Despite the standard operating procedures were required when handling vomitus, some cleaners and teachers fail to adhere to them, which has resulted in inconsistent cleaning quality between various classes, which included the use of emesis bags and timely cleaning and disinfection for contaminated area.

We conducted a retrospective cohort study to examine the risk factors for diarrhea or vomiting. Suspected cases were defined as events involving vomiting or diarrhea among employees, students, and their respective family members since February 1, 2023. Clinical cases were defined as suspected cases exhibiting diarrhea more than 3 times within a 24-h period. The clinical cases with PCR positive for sapovirus nucleic acid were defined as confirmed cases. We administered a structured questionnaire by telephone or in person to all the employees, students and their family members, recording demographic information and characteristics of the illness. This study was approved by the Ethics Committee of SBCDC.

### Environments inspect

Each floor of the building has a single washing room that can accommodate 5 boys and 7 girls simultaneously. But the number of students and teachers in every floor exceeds 290. Every washing room has two sinks equipped with touchless sensor faucet. There is no fixed schedule for washing room disinfection and standard operation procedure for vomit disposal. Emesis bags were not always provided for sick students in classroom. Several cases described seeing vomit in various areas, such as in restrooms, hallways, and classrooms.

### Laboratory investigation

A total of 11 stool specimens were collected from students with AGE and then sent to SBCDC laboratory. The nucleic acids of norovirus and sapovirus were tested using commercial real-time RT-PCR kit (zybio Limited company, Chongqing, China), and rotavirus using real-time RT-PCR kit (bioPerfectus technologies limited company, Taizhou, China) according to the instructions provided by the company, respectively. The nucleic acids of bacteria and toxin genes were detected using FilmArray GI panel on Biofire^®^FilmArray^®^ system (bioMérieux, Marcy I'Etoile, France). The bacteria include Campylobacter (jejuni, coli and upsaliensis), Plesiomonas shigelloides, Salmonella, Yersinia enterocolitica, Vibrio (parahaemolyticus, vulnificus and chloerae), Vibrio cholera, Enteroaggregative E. coli (EAEC), Enteropathogenic E. coli (EPEC), Enterotoxigenic E. coli (ETEC) It/st, Shigella/Enteroinvasive E. coli (EIEC), E. coli O157. Toxin genes include Shiga-like toxin genes 1 and 2 (stx1/stx2) and Clostridium difficile (Toxin A/B).

### Statistical analysis

Attack rate was calculated as the proportion of cases among the targeted students in the school. The data sorting and drawing were conducted by Excel 2007, and the database was established by Epidata 3.1. Comparisons of categorical data were performed using Pearson's Chi-square test or Fisher's exact test and Chi-square for trend with distance from vomitus. The odds ratio (OR) was calculated. A *p*-value of <0.05 was considered statistically significant. All statistical analyses were performed with R 3.5.3 software (R foundation for statistical computing, Vienna, Austria).

## Results

### Descriptive epidemiology

**Figure 2:**
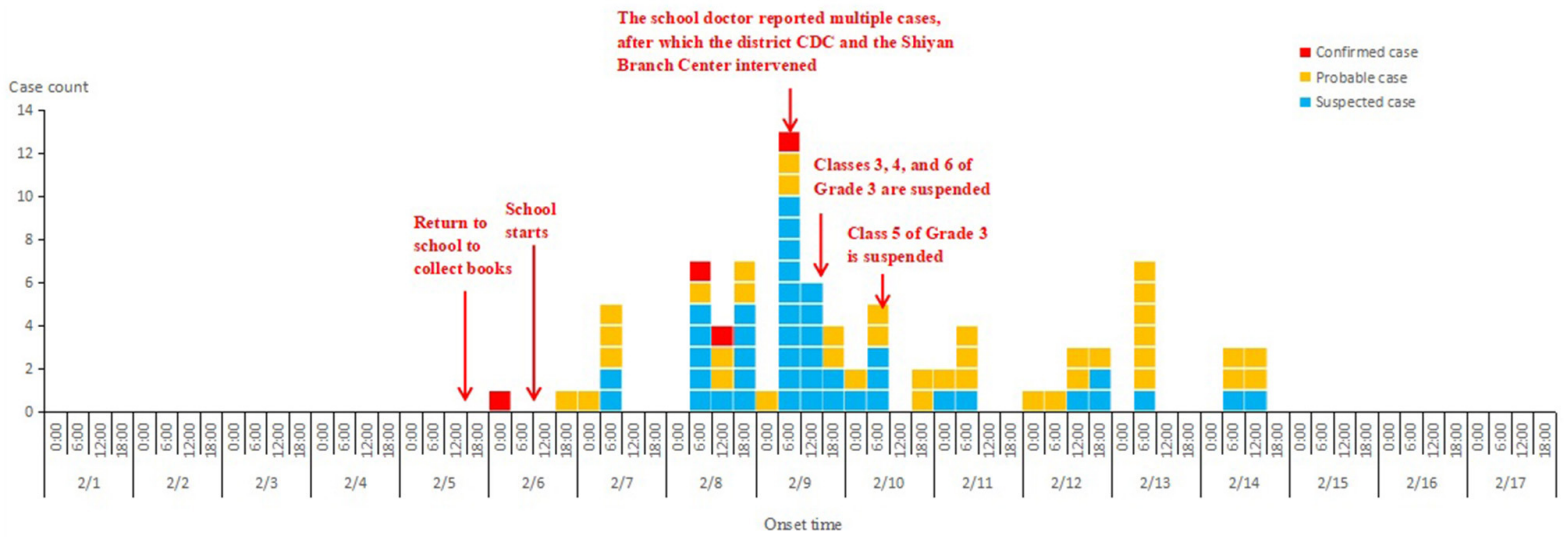
Date of onset of acute gastroenteritis symptoms observed in a school, Bao'an district, Shenzhen, China, February 2023.

A total of 436 questionnaires were distributed to all the students of 9 classes on the third floor of Building 2, with 423 being returned, resulting in a response rate of 97%. Of these, 386 were valid, yielding a validity rate of 90.1%. A total of 86 suspected cases were identified in this outbreak, including 85 students and one teacher. 65.1% were male, 34.9% female. 91.9% of them were elementary school student students, 7.0% were middle school students. 70.9% of them reported vomiting, 53.5% diarrhea, 38.4% bellyache, 14.0% feel nauseous (Table 1). 46.6% of cases experienced diarrhea only, while 29.1% had vomit only. Both of vomiting and diarrhea were reported in 24.3% of cases. Among students with vomiting, 77.0% of them vomited no more than twice, 16.4% vomited three or four times. Among students with diarrhea, 69.6% of them had diarrhea no more than twice, and 26.1% had diarrhea three or four times. This outbreak lasted for 9 days with case numbers peaking at the fourth day. It peaked at 6:00 a.m. on February 9 and ended at 12:00 on February 14. 65.1% cases were reported from building 2, followed by building 5 with 11.6% cases (Table 2). A teacher had diarrhea at 8:00 p.m. on February 8th and then asked for a leave of 5 days. The corresponding number of cases at different classrooms and offices were also shown. 53.5% of cases (46/86) were from the third floor of building 2. Among cases in building 2, 82.1% (46/56)of them were from the third floor (Table 3). The first case appeared at 5:00 on February 6, the number of cases peaked between 8:00 and 12:00 on February 9. The third, fourth, and sixth class of third grade are suspended from 12:00 on February 9 to 8:00 on February 13 due to acute gastroenteritis outbreak. Afterwards, the number of reported suspected cases declined (Figure 2).

**Table 1:** The clinical symptom profile of 86 cases in this AGE outbreak in Bao'an district, Shenzhen, China, February 2023.

**Symptoms**	**Number**	**Proportion (%)**
Vomit	61	70.9
**The number uun of vomit per day**		
1–2	47	77.0^a^
3–4	10	16.4^a^
5–6	2	3.3^a^
7–8	0	-
9–10	2	3.3^a^
**Diarrhea**		
The number of diarrhea per day	46	53.5
1–2	32	69.6^b^
3–4	12	26.1^b^
5–6	1	2.2^b^
7–8	1	2.2^b^
Abdominal pain	33	38.4
Sicchasia	12	14.0
Headache	5	5.8
Abdominal distension	2	2.3
Fever	2	2.3
Megrim	1	1.2

^a^the proportion is calculated using the number of cases having vomit as denominator.

^b^the proportion is calculated using the number of cases having diarrhea as denominator.

**Table 2:** The incidence of AGE in this outbreak at various buildings at the school in Bao'an District, Shenzhen, China, February 2023.

**Building No**.	**Case**	**The number of student**	**Incidence (%)**	**Chi-square**	***P***
1	6	241	7	11.7	0.02
2	56	633	65.1		
3	6	98	7		
4	7	142	8.1		
5	10	191	11.6		

**Table 3:** The incidence of AGE in this outbreak at the school in Bao'an district, Shenzhen, China, February 2023.

**Floor**	**Case**	**Number of student**	**%**	**Chi-square**	***p*-value**
3	46	168	27.4	361.2	<0.001
2	5	148	3.4		
1	2	100	2		
4	2	49	4.1		
5	1	51	2		

### Risk factors for this AGE outbreak

In the retrospective cohort study, eating food provided by school or drinking water from direct-drinking machine was not associated with the acquisition of AGE based on univariate analysis (Table 4). Went to washing room at the third floor of building 2 is also not a risk factor with AGE. The students who had passed near a spot of vomit (<2.4 m) were more likely to get AGE with a relative risk of 5.09 (95%CI: 2.58, 10.04). To confirm the impact of the distance from vomit to student when their passing near vomit, we compared the incidence of AGE between two group of students passed near vomit less than and more than 1.2 meters (Table 5). Those students with a distance <1.2 meters from vomit are more likely get AGE with RR value of 1.94 (95%CI: 1.06, 3.55; Table 5). The case number with AGE in classrooms using standard operating procedure for vomit cleanup is obviously lower than that in ones not handling vomit according to the standard procedure (RR: 0.34, 95%CI: 0.15, 0.77; Table 6).

**Table 4:** Univariate analysis of risk factors involved in this outbreak of AGE in Bao'an district, Shenzhen, China, February 2023.

**Variable**	**Exposed**		**Unexposed**		**Incidence(%)**		**χ2**	***P***	**RR (95%CI)**
	**cases**	**Total**	**cases**	**Total**	**Exposed**	**Unexposed**			
Passed near a spot of vomit (<2.4 m)	32	147	10	234	21.77	4.27	28.17	<0.05	5.09 (2.58, 0.04)
Went to washing room at the third floor on building 2	40	332	2	49	12.05	4.08	2.74	>0.05	2.95 (0.74, 11.83)
Eating food provided by school	29	210	13	171	13.81	7.60	3.70	>0.05	1.82 (0.96, 3.38)
Consume direct drink water	20	158	22	223	12.66	9.87	0.74	>0.05	1.28 (0.73, 2.27)

**Table 5:** The contribution of passed vomitus within <1.2 meters to the onset of AGE outbreak in Bao'an district, Shenzhen, China, February 2023.

**Passed vomitus within <1.2 meters**	**Total number**	**Case**	**Incidence (%)**	**χ2**	***P***	**RR (95%CI)**
Yes	50	16	32.00	4.66	<0.05	1.94 (1.06, 3.55)
No	97	16	16.49			

**Table 6:** The contribution of cleaning vomit using standard operating procedure to the onset of AGE in this outbreak at the school in Bao'an district, Shenzhen, China, February 2023.

**Cleaning vomit using standard operating procedure**	**Student number**	**cases**	**Incidence (%)**	**χ^2^**	***P***	**RR (95%CI)**
Yes	43	6	13.9	7.99	<0.05	0.34 (0.15, 0.77)
No	41	17	41.46			

### Laboratory results

Four out of 11 stool specimens were positive for sapovirus nucleic acid while negative for norovirus and rotavirus nucleic acid. None of them were positive for bacteria and toxin genes mentioned above.

### Control measure

Firstly, three classes were suspended from the afternoon of February 9th, and one from February 10th. The suspension will last for at least 3 days. Secondly, the school enhanced the implementation of morning checks for students attending school. Students having a fever, diarrhea, or vomiting during the morning health check will be sent home to see a doctor. All other students and staff will be monitored over 1 week. All classrooms, restrooms, and stairwells were subjected to a twice-daily disinfection regimen for a week.

## Discussion

In this study, our findings indicate that sapovirus was the etiological agent contributing to this acute gastroenteritis outbreak. Although sapovirus is one of the leading causes of acute viral gastroenteritis globally, second only to norovirus in some countries, AGE outbreaks caused by sapovirus in China were reported infrequently. Generally, the symptoms of AGE caused by sapovirus are milder than those by norovirus (15, 16). As in this study, the times of diarrhea and vomit and the incidence of abdominal pain were far lower compared with AGE caused by norovirus (13). Therefore, AGE caused by sapovirus may be more likely ignored. Up to now, <15 AGE outbreaks associated with sapovirus occurred in mainland China (17–19). Among them, food was determined an infectious source in only two outbreaks while it was not defined in the other outbreaks (20). Although person-to-person transmission was the major spread mode, almost account for one-third of these outbreaks, there was no strong epidemiology evidence to support these inferences, even an index case was not found in most outbreaks. In this outbreak, it is suspected that the index case acquired a viral infection from the consumption of contaminated food, and the incubation time and symptom was accordance with sapovirus AGE.

The fecal-oral route, often through contaminated food or water, is the primary transmission mode for the acute gastroenteritis outbreak caused by sapovirus (10). Among secondary cases, person-to-person transmission is the most significant mode of spread (3, 10). Although only several reports suggest airborne transmission as a major route during outbreaks of acute viral gastroenteritis caused by sapovirus, most of them lack sufficient epidemiological evidence (1, 20). In this outbreak, neither eating food nor drinking water in school did not increase the risk of getting AGE. Only being near vomit was associated with a 4.09-fold increase in the risk of getting AGE (RR of 5.09, 95%CI: 2.58–10.44). A strong correlation exists between the risk of infection and the distance from vomitus. A contact distance of <1.2 meters posed a significantly higher risk of infection compared to a distance of <2.4 meters. Our results are accordance with Chen's study where the distance of <1 meter increase the risk of getting sapovirus infection (21). In general, when sharing a classroom or washing room with the students having AGE, it is difficult to keep away their excrete. But keeping an appropriate distance from excrete may be practicable for students and staff in a school.

Emesis is important in terms of riding the upper gastrointestinal tract of toxins and pathogens. Previous study revealed that splashes and droplets produced during an episode of projectile vomiting can travel great distance of more than 3 meters forward spread and 2.6 meters lateral spread (22, 23). Therefore, cleaning and decontamination of surfaces contaminated with an infectious agent post-emesis is therefore an important aspect of infection control. In our study, we found that cleaning and disinfecting vomitus in time and using emesis bag during vomit decrease AGE incidence (Table 3). As an emergency measure to curb the spread of infectious diseases in schools, the suspension of face-to-face classes is an effective measure to mitigate transmission of virus infection in children (24, 25). Compared to school closure or suspension, it is easier to require school cleaning workers using disposal emesis bag during students' vomiting and clean up and disinfect vomitus and excrete immediately.

There is some limitations. Despite the suspicion that the index patient's illness was caused by the dumplings he consumed, no samples from the same batch were collected for sapovirus test. During our investigation, we discovered that only the index case ate the potential contaminated dumplings while his parents had not. In this family, only index case had AGE. This supports that dumpling was the original infectious source together with incubation time of 12 h after had dumplings. In addition, we did not identify genotype of sapovirus and test the carriage rate of sapovirus among asymptomatic population.

While airborne transmission has been recognized as a significant route of calicivirus spread, robust epidemiological evidence from outbreak studies remains limited. Our research further emphasizes the importance of airborne transmission in sapovirus outbreaks. Providing emesis bags to ill students and promptly and properly cleaning contaminated areas can significantly reduce the incidence of acute viral gastroenteritis. These measures should be considered crucial for schools in preventing and controlling AGE and other infectious diseases.

## Conclusion

Our research identified that sapovirus was the causative agent of this AGE outbreak. Airborne transmission was the primary route of infection. Prompt decontamination and the use of vomit bags significantly reduce the incidence of AGE while close contact with contaminated materials increases the risk of infection. These measures should be prioritized in public health strategies for the prevention and control of viral gastroenteritis outbreaks.

## Data Availability

The raw data supporting the conclusions of this article will be made available by the authors, without undue reservation.
